# Everybody's Page

**Published:** 1890-11-01

**Authors:** 


### Everybody's Page.

There are twelve Epileptic Institutions in Prussia which have all been erected by private generosity.

The life of the first Napoleon was regarded in the year 1804 as so uncertain that a premium of 55 guineas per cent. was paid upon it at that time for one year.

The Congo savages are not lacking in shrewdness. Mr. Ward one day said to a native, who brought him a fish which was repulsively stale, "White men don't eat fish that smell like that." "But," answered the native, "you don't eat the smell!"

Naphthol, A. 20 centigrammes, distilled water 1,000 grammes, is recommended in the treatment of purulent ophthalmia by Dr. Budin, of the Paris Charity Hospital, his opinion is that with naphthol the swelling of the eyelids rapidly yields, and that it is infinitely preferable to boric water.

It has been suggested by the Lower Austrian Diet that drunkards' homes shall be established for curative purposes. This suggestion is opposed by the Austrian Sanitary Board on the ground that it is very doubtful whether alcoholism is curable, and that the probability of completely rescuing habitual drunkards is so small as not to warrant legislation and heavy expenditure. What will temperance people say to this ultimatum?

The New York *Medical Record* says that if the returns of the number of patients treated at the dispensaries of New York are correct, the number of medical paupers of that city have increased in two years from 350,000 to nearly half-a-million! The *Record* asks, rather sadly, "Is this charity?" These returns mean that one out of every three or four inhabitants receives medical advice gratis, which looks as though charity were sometimes misappropriated across the pond.

That was a terrible case of diphtheria outbreak at Cheam, Surrey, which came before the Kingston Rural Sanitary Authority last Thursday. Some of the cottages in this village are quite unfit for human habitation, and as there were no means at all of isolation, the disease could not but spread. The sick, dying, and dead were all huddled together, because their own sanitary (?) authority (Epsom) could give them no help in the way of isolation. It seems a preposterous thing that in these days any sort of isolation accommodation should be lacking. It says little for the public spirit of the neighbourhood, but it shows to what an alarming extent apathy regarding disease prevention still prevails.

It certainly seems necessary for those who are victimised by the constant receipt of begging letters to be very careful what they do with such requests. Though a very large proportion of these documents are from the pen of impostors and people who would not earn an honest livelihood if it were within their grasp, a few, but, alas! very few, are written by more or less deserving objects. It may be alleged, and not without justice, that a person who descends to writing a begging letter to a perfect stranger is so lost to all sense of self-respect and self-reliance as to be unworthy of assistance. Certainly assistance should not be given, as undoubtedly it frequently is, without full inquiry. But how can any man, especially a busy man, who is usually the selected victim, find time to look into the deserts of an average of three letters a-day, representing demands for specific sums amounting to over £3,000! This sum is what the Bishop of London calculates he is asked to disburse in the course of the year.

It is said that in the year 1368 to 1369, the Hotel Dieu in Paris buried 22,000 of its patients; the average duration of life in the middle ages was not more than seventeen years.

During the past four or five years some ingenious manufacturers in Paris have been selling some water mixed with various salts as natural mineral waters. The suspicions of certain doctors being aroused by the unpleasant effects resulting to their patients from the use of some of these waters led to an analysis of them and to the discovery of the fraud.

Owing to the greater attention paid to hygiene and sanitary matters generally the mortality among French soldiers in their colonies has shown a very remarkable diminution in the past thirty years. Where the death rate during the earlier part of the century was as high as 77 per thousand, it has now been reduced to as low as 11 or 12 per thousand.

Kensington inhabitants are about to combine against the "smells," which irate householders have been complaining of in the dailies just lately. It is a comfort to find that some, at least, realise that union in these matters is the only way to achieve reform. The individual protest effects very little, but we hope that the effort of an entire parish will raise a voice too strong to be stifled.

"These 'ere carriages," remarked a grumbly masculine voice in the next compartment to us of a third class carriage on the underground railway, "were built for us working men, they're ours by right, the drones have no right to get into 'em; the drones get a great deal more as it is than they ought to. Now look at a man like Lord Rothschild, wot does he want with fourteen bedrooms, and a hundred pair of trousers? He can't want fourteen bedrooms, and he can't wear more than one pair of trousers at a time, and so ninety-nine other men have to be without trousers all through 'im." And just as the masculine voice arrived at this logical conclusion he arrived at his destination. But we feel that the 3rd class carriage is undoubtedly the place to pick up information.

The Narghilé, or water-pipe, commonly supposed to be a harmless method of tobacco indulgence, has been the subject of investigation by Dr. Emirzé of the Armenian Hospital at Smyrna. The Narghilé has a bowl for the tobacco, and from this a tube extends into a vessel containing about two quarts of water; to this is fixed a flexible tube with the mouthpiece, and the smoker draws the smoke through the water. Dr. Emirzé finds that the effect of the Narghilé, unless used with the utmost moderation, is quite as bad as that of cigarettes or cigars, and it has an additional bad result caused by the inspiratory effort necessary to draw the smoke through the water. Smoked once or twice daily, Dr. Emirzé considers the Narghilé might prove beneficial as a respiratory exercise, but used in any excess it induces chronic pharyngeal, laryngeal, and bronchial catarrhs, and a host of other evils. What then is the smoker to do—his cigar, his cigarette, the homely pipe, and now the hubble-bubble are all condemned? There remains the only consolation that he must enjoy the fragrant weed in the greatest moderation or suffer. John Locke, who was sedate philosopher enough, says: "Bread or tobacco may be neglected, but reason first recommends their trial, and custom makes them pleasant," but if his ghost walks around now he must, we think, consider that custom has made tobacco a little too pleasant.

